# An Innovative Approach for Facial Rejuvenation and Contouring Injections in Asian Patients

**DOI:** 10.1093/asjof/ojaa053

**Published:** 2021-02-13

**Authors:** Haiyan Cui, Haiguang Zhao, Haisong Xu, Guobao Wang, Linlin Tan

**Affiliations:** 1 Department of Plastic and Cosmetic Surgery, Tongji Hospital, Tongji University School of Medicine, Shanghai, China; 2 Plastic and Aesthetic Surgery Department of Huadong Hospital Affiliated to Fudan University, Shanghai, China; 3 Department of Plastic and Reconstructive Surgery, Shanghai Ninth People’s Hospital, School of Medicine, Shanghai Jiao Tong University, Shanghai, China; 4 Hangzhou Time Plastic and Cosmetology Hospital, Hangzhou, China

## Abstract

An increasing number of Asian people are seeking nonsurgical facial aesthetic treatments. Ethnic Asians differ from Western populations in both facial appearance and baseline structural facial anatomy. And there is a lack of clinical instruction to doctors who provide facial aesthetic treatment for Asian patients. The authors proposed the “未来 Future Codes” design in Chinese calligraphy describing the art of facial injection in Asians to help doctors perform well. “未来” are pictograph of 2 Chinese characters, translated into English as “Future,” which represent beautiful meanings and vividly describe the procedure and operating area of the design methods. The concept encompasses a systematic overall design for the art of facial injection in Asians, and these procedures are easy to learn and perform safely. This is the first systematic solution available in the clinic that can be used to design facial aesthetics and rejuvenation in Asians through Eastern philosophy and culture.

**Level of Evidence: 5:**

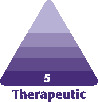

In the past decade, there has been an increase in nonsurgical cosmetic procedures worldwide. Many patients choose dermal fillers and botulinum toxin to alter their appearance and restore tissue loss.^1^

Botulinum toxin and dermal fillers are the 2 most popular nonsurgical cosmetic procedures performed globally to treat age-associated changes and are often used in combination.^2^ There are many kinds of filler materials for volume replacement and enhancement, such as hyaluronic acid (HA),^3^ collagen, poly-l-lactic acid,^4^ and calcium hydroxylapatite.^5,6^ These fillers can be used for cheek and chin augmentation, nose reshaping, midface volumization, and lip enhancement. To reduce the appearance of wrinkles, many people choose injections of botulinum toxin. Botulinum toxin relaxes certain muscles on the face to make wrinkles less noticeable for a period of time.^7^

The use of nonsurgical aesthetic facial treatments is also increasing in Asia.^7^ To date, most of the studies of facial injection therapy have evaluated Western populations, although Asian populations differ in terms of culture, anatomical structure, perceptions of beauty, and signs of aging.^8,9^ For the above reasons, there are many differences in desired outcomes between Caucasians and Asians. A thorough understanding of the key aesthetic concerns and requirements for the Asian face is required to instruct appropriate facial aesthetic treatments. Existing published guidelines cannot be directly applied to Asians. Thus, recommendations that are suitable for Asian populations are needed.

Most of the Asian patients, regardless of age, prefer to avoid surgery as much as possible and seek natural results. Therefore, patients’ expectations should be evaluated, and innovative procedures should be proposed to meet Asian patients’ aesthetic needs, including the facial shape, structure, and proportion, as well as the effects of aging on their faces. The aesthetic standards of Eastern and Western populations remain different. Westerners possess clear facial contours, narrow cheekbones, contouring structures, and obvious light and shadow effects. Oriental faces are plump, with large cheekbones, unclear contours, and delicate skin (Figure 1). Facial filling is commonly performed to restore vitality and resist aging in Western countries. Orientals focus on filling and contouring. As Asians differ from Westerners in facial appearance and anatomical structure, noninvasive aesthetic treatments are appropriate for Asians.^7^ However, to date, most of the studies and recommendations regarding facial injection have referred to Western populations.^10-12^ Regardless of national and cultural differences, certain aesthetic principles, including symmetry, balance, proportion, coordination, and unity of diversity, are shared in common between Eastern and Western populations (Figure 1). In addition to these basic principles, the following features are also commonly considered to be pleasing: (1) elegant bearing, (2) slim figure, (3) beautiful face, (4) slender neck, (5) proportional breasts, (6) narrow waist, and (7) round hips.^7,13^ The highest level of aesthetic injection treatment serves not just as a simple filler injection procedure but also as an artistic creation under the limitations of medicine.

**Figure 1.**
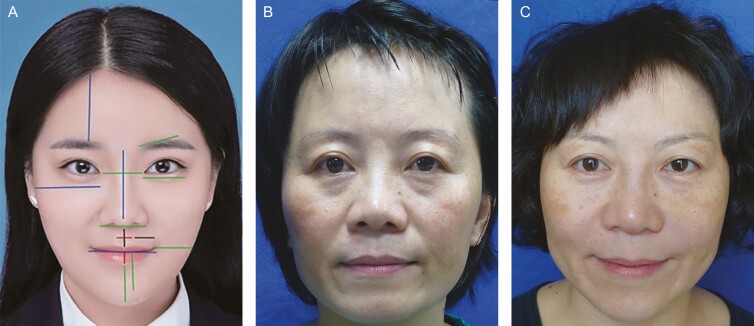
(A) The proportion markers on a beautiful Asian face (26-year-old female). The green line is the baseline X, then the blue line is 1.618 X, the black line is 0.618 X, the orange line is 0.382 X, and all the same color lines are the same length; 1.618, 0.618, and 0.382 are all gold ratios (the theory of proportion markers from Dr Arthur Swift).^14^ (B, C) Female, 48 years old, before (lower left) and 3 months after injection (lower right) according to “Cui Codes” design method.

Researchers have discovered that the human perception of physical beauty is closely related to the golden ratio (as rounded to 1.618). The ratio of the distance between certain regions of the face relative to the distance of another defined region is considered ideal at 1.618.^15^

Most of the faces regarded as beautiful or attractive have a significant number of markers whose proportions are very close to the golden ratio. As shown in Figure 1B, the green line is the baseline X, the blue line is 1.618 X, the black line is 0.618 X, and the orange line is 0.382 X; lines of the same color are the same length; 1.618, 0.618, and 0.382 are all gold ratios, and there are golden ratios between various lines. Therefore, proportional aesthetics are an important feature of beauty and occupy an important position in formal aesthetics.^14^

The authors^16^ published a paper in 2007 putting forward the concept of overall aesthetic design, which includes a systematic overall design and aesthetic evaluation before the treatment procedure, psychological counseling during the peri-treatment period, and the comprehensive use of multiple methods. After treatment, making up, clothing, modeling, and etiquette training are necessary complements to create a beautiful appearance that would be deemed to exude charm and vitality.

In the authors’ opinion, cosmetic doctors can be divided into 3 levels as follows: the ones are on the first level who use various products and equipment, just master basic injection techniques and basic knowledge of anatomy to solve basic problems. At the second level, those are the ones who are experienced in facial injection and can prevent and manage complications. At the third level, mastering the principal injection techniques and deepening their fundamental knowledge, doctors regard injection therapy as an artistic creation conducted under medical restrictions in order to create beauty in individuals; the creation of beauty will be achieved through systematic and overall design instead of a single method of treatment.

As a biopsychosocial medical model is advocated, physicians’ aesthetic perception and humanistic care are required in addition to surgical techniques, especially in the field of plastic surgery. Cosmetic injection should follow the principle of the overall design. Many patients have reported being unsatisfied with results, even if the operations were performed very well, because their doctor only solved the focal problem and neglected the relationships between the parts and the whole.^7,13^ For instance, a perfect nose must have a good proportional aesthetic relationship with the entire face, as well as a patient who originally intended to correct his or her nasolabial folds. After the injection, the nasolabial fold problem was solved, but the face became fat. Such cases demonstrate that it is particularly important to improve aesthetic perception and humanistic care, especially among cosmetic surgeons.^17^ Before performing a cosmetic injection, the overall design and construction of the human aesthetic image must be considered, which will lead to a better therapeutic outcome, psychological satisfaction, and social recognition of the patients. Furthermore, care should be taken to maintain the patient’s psychological state throughout the treatment according to the biopsychosocial medical model.

In the summary of thousands of teaching and training cases, the authors proposed the “未来 Future Codes” design in Chinese calligraphy describing the art of facial injection in Asians in order to help doctors perform well. “未来” (Figure 2A) are pictograph of 2 Chinese characters, translated into English as “Future,” which represent beautiful meanings and vividly describe the procedure and operating area of the design methods. The “Future Codes” describe a concept of the systematic overall design that is easy to learn and can help doctors obtain a satisfying result. The injection method in this article is hidden in the Chinese calligraphy of the 2 characters, consisting of the following lines and reflecting the aging characteristics of the Asian face (Video).

**Figure 2.**
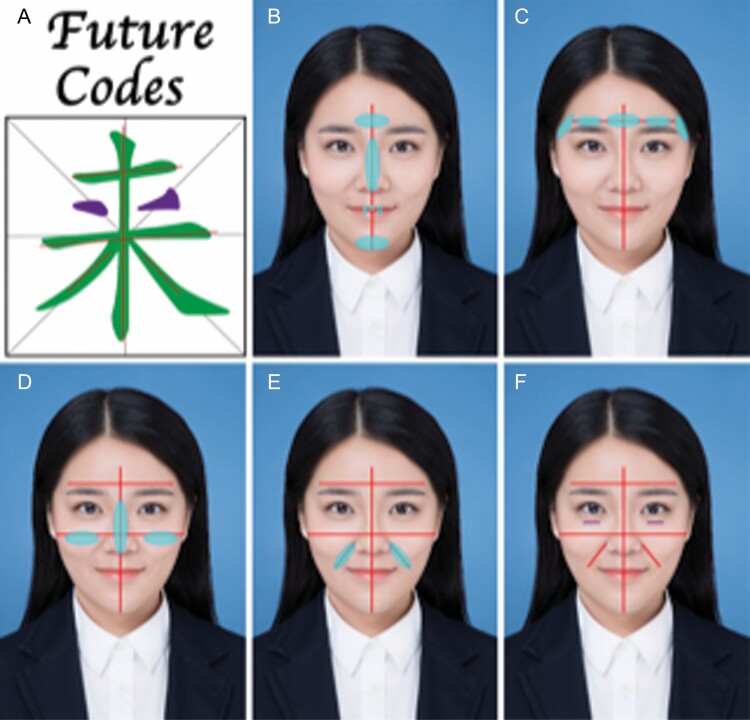
Dr. Cui’s “Cui Codes” design based on Chinese calligraphy. (A) 2 Chinese characters “未来,” which means “Future” in English. The concept encompasses the systematic overall design for the art of facial injection in Asians. (B) The middle line of the face (a 26-year-old female) passes through the forehead, the glabella complex, the nose, the lips, and the chin. (C) The first horizontal line passes the arch of the temper region, the eyebrow, and the glabella complex. (D) The second horizontal line passes through the cheeks or the “apple muscle.” (E) The other 2 oblique lines run along the nasolabial folds. (F) Finally, 2 nasojugular folds, tear trough, are added.

The middle line of the face (Figure 2B) passes through the forehead, the glabella complex, the nose, the lips, and the chin. This line is highly important and can affect the success or failure of the whole procedure because it determines the contour, symmetry, balance, coordination, proportion, stereo, light, and shadow on both sides of the face. Therefore, this is a key line in the “Future Codes” design.

The first horizontal line (Figure 2C) passes the arch of the temper region, the eyebrow, and the glabella complex. The second horizontal line (Figure 2D) passes through the cheeks or the “apple muscle.” If an injection is applied inside this line, the cheekbone will appear narrower, helping reduce the width of the cheekbones in Asians. The horizontal lines of the eyebrow and the “apple muscle” are also considered as aging criteria. Obvious lacrimal sulcus and nasolabial folds are considered as signs of aging. On the other hand, the other 2 oblique lines run along the nasolabial folds (Figure 2E). The patient will appear younger if the nasolabial folds are treated with injection fillers. Finally, 2 nasojugal folds and the tear trough are added (Figure 2F), which combine the 2 Chinese characters and the “Future Codes.” The “Future Codes” design includes the important aesthetic points and lines of facial contour and encompasses the core principles of facial rejuvenation and aesthetics (Figure 2). All these lines make up the word “Future” in Chinese characters. Therefore, the Chinese calligraphy of the 2 characters represents the core of aesthetic facial injections in Asian patients.

The appearance of aging is mainly due to relaxation of the skin and soft tissue, displacement of the tissue structure, the appearance of skin folds, changes in the texture of the skin, and the lack of tissue capacity and elasticity. These features are also reflected in the “Future Codes.” The operation and injections are feasibly performed with knowledge of anatomy, and complications can be effectively avoided by following this injection protocol.

## CASE DEMONSTRATION

A 48-year-old female underwent the injection process according to the authors’ “Future Codes” design method:

1. Injection at the middle line: (1) The glabella complex is injected with 1 mL HA by puncturing with a sharp needle until the tip touches the periosteum, ensuring no blood is withdrawn, and then pushing slowly. The area is massaged while injecting. (2) The nose is injected with 1 mL HA plus 1% lidocaine for local anesthesia by puncturing the tip of the nose with a 23G blunt needle. (3) The lips are injected with a total of 0.4 mL HA with a 30G sharp needle: 3 points on the upper lip and 2 points on the lower lip are injected with 0.08 mL each. (4) The chin is injected with 1 mL HA with a 27–30G sharp needle by puncturing with the sharp needle until the tip reaches the periosteum.
2. Injection of the first horizontal line: (1) The eyebrows are injected with 0.6 mL HA with a 23G blunt needle by subcutaneously puncturing with a blunt needle at the eyebrow tail. HA is injected into the eyebrow while subcutaneously retracting the needle. (2) The temporal region is injected with 2 mL HA using a sharp needle by touching the needle tip to the periosteum, ensuring no blood is withdrawn, and then pushing slowly. Each side of the temporal region is injected with 1 mL HA while massaging.
3. The second horizontal line for the “apple muscle” is injected with 1 mL HA. For the apple muscle, the nasolabial folds, and the tear trough, a 23G blunt needle is used to puncture 1.5 cm away from the outer corner of the mouth, subcutaneously or on the periosteum, and 0.8 mL HA is injected on each side. The needle tip can reach the apple muscle, the tear trough, and the nasolabial folds.
4. The nasolabial folds are injected with 2 mL HA by puncturing 1.5 cm away from the outer corner of the mouth, with 1 mL injected on each side.
5. The tear trough is injected with 0.3 mL HA by puncturing 1.5 cm away from the outer corner of the mouth, with 0.15 mL injected on each side.

### Adjuvant therapy

After injection treatment, the following procedures can also be applied to achieve better effects: (1) a fiber laser can be used to dissolve eye bag fat; (2) radiofrequency can be used to tighten the skin of the lateral corner of the mouth and the mandibular margin; and (3) hair can be trimmed.

### Result

Through the systematic comprehensive “Future Codes” design, the patient acquired a face with harmonious ratios, a contouring structure, clear light and dark contrast, tightened skin, and flattened eye bags. The procedures resulted in the appearance of more spirited eyes, a stereo and natural nose structure, rounded apple muscles, narrow cheekbones, shallow and round nasolabial folds, and enhanced length, convexity, and radius of the chin. The entire facial appearance was perceived as younger (Figure 1D, Supplemental Figures 1 and 2).

## STATEMENT

The ethical approval of the case demonstration was given by the medical ethics committee of Tongji Hospital Institutional Review Board, Tongji University, Shanghai. And the written informed consent was obtained for each participant according to federal and institutional guidelines.

## CONCLUSIONS

An increasing number of Asian people are seeking nonsurgical facial aesthetic treatments. Ethnic Asians differ from Western populations in both facial appearance and baseline structural facial anatomy. However, there is a lack of clinical instruction to doctors who provide facial aesthetic treatment for Asian patients. Therefore, there is an urgent need for instruction to guide physicians in performing cosmetic injection treatments for Asian patients, and these procedures should be easy to learn and perform safely. The authors’ “Future Codes” design is derived from the Chinese calligraphy of 2 Chinese characters, which means “Future” in English. The concept encompasses a systematic overall design for the art of facial injection in Asians. The author H.C. has more than 10,000 cases of treatment experience and has extensive teaching and training experience. This method is associated with beautiful meanings and is easy for clinicians to master. This is the first systematic solution available in the clinic that can be used to design facial aesthetics and rejuvenation in Asians through Eastern philosophy and culture.

## Supplemental Material

This article contains supplemental material located online at www.asjopenforum.com.

## Supplementary Material

ojaa053_suppl_Supplementary_Figures
