# Supplementary material for: An Innovative Approach for Facial Rejuvenation and Contouring Injections in Asian Patients
Source: Aesthet Surg J Open Forum. 2021 Feb 13;3(2):ojaa053. doi: 10.1093/asjof/ojaa053 (PMC8240745; doi:10.1093/asjof/ojaa053)

**Supplemental Figures**

**Supplemental Figure 1.** A 37-year-old female underwent the injection process according to the authors’ “Future Codes” design method, (A) preoperative and (B) 2 weeks postoperative.


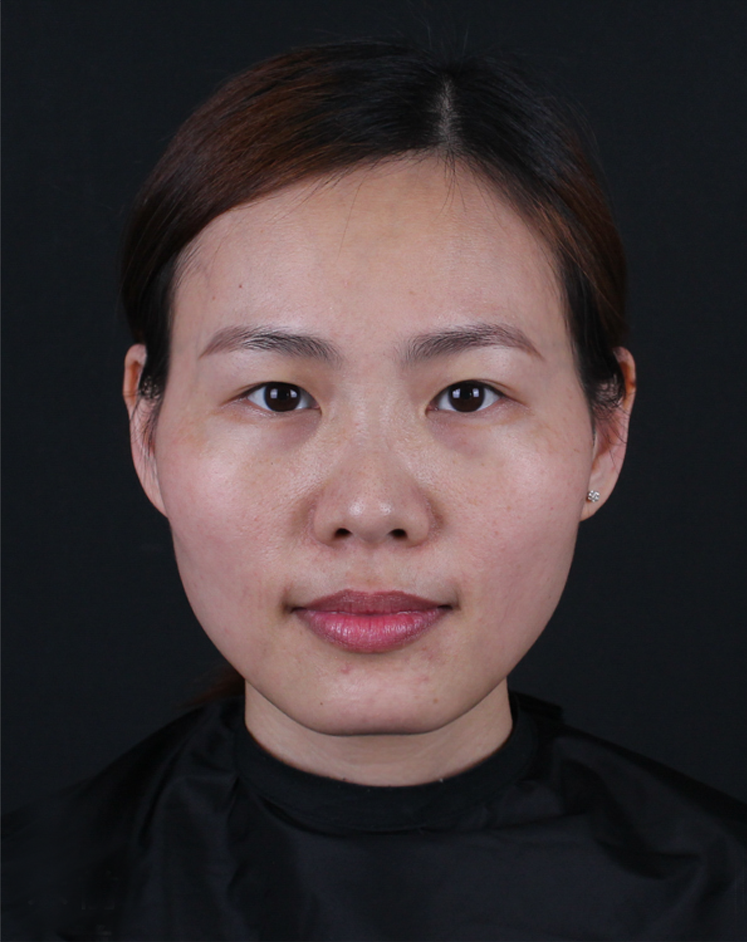

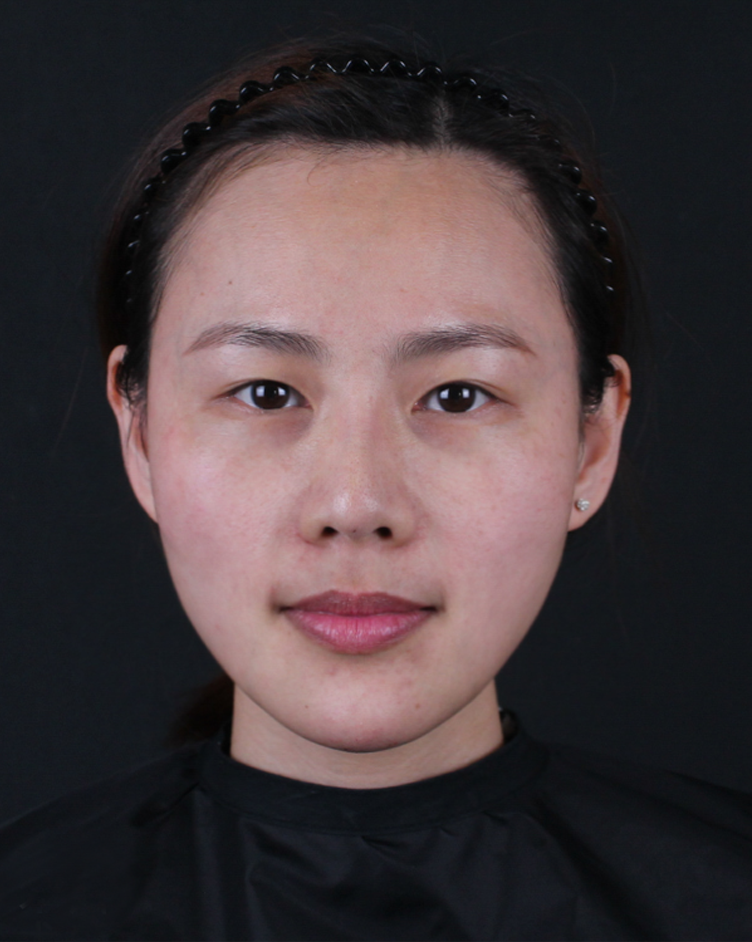


**Supplemental Figure 2.** A 42-year-old female underwent the injection process according to the authors’ “Future Codes” design method, (A) preoperative and (B) 1 week postoperative.


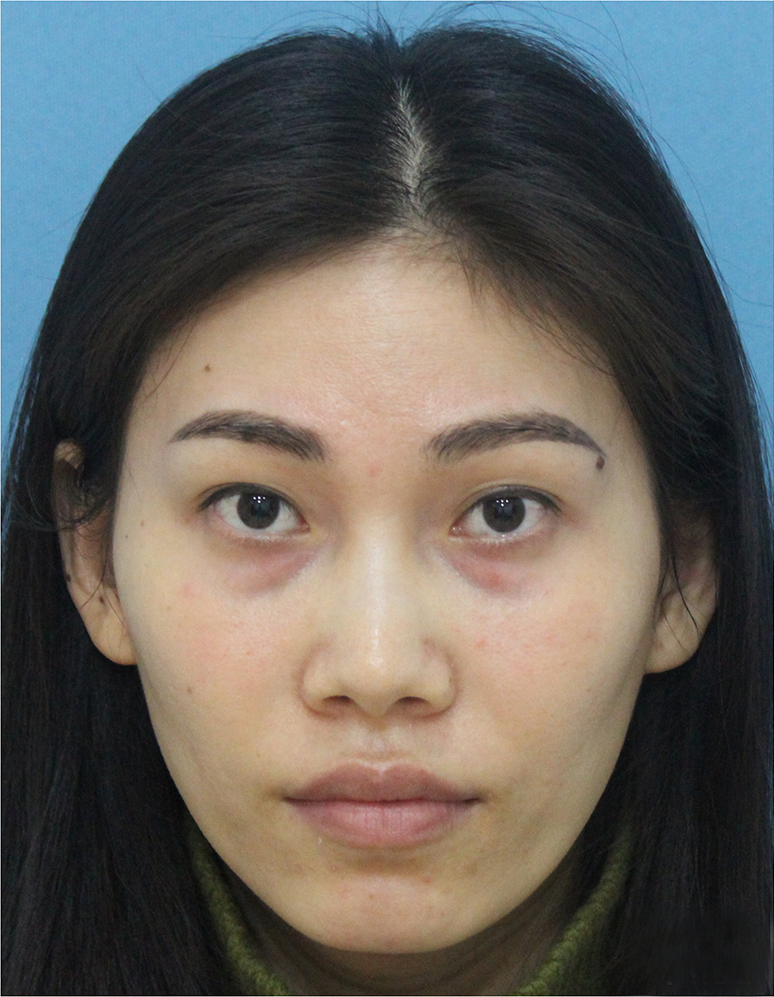

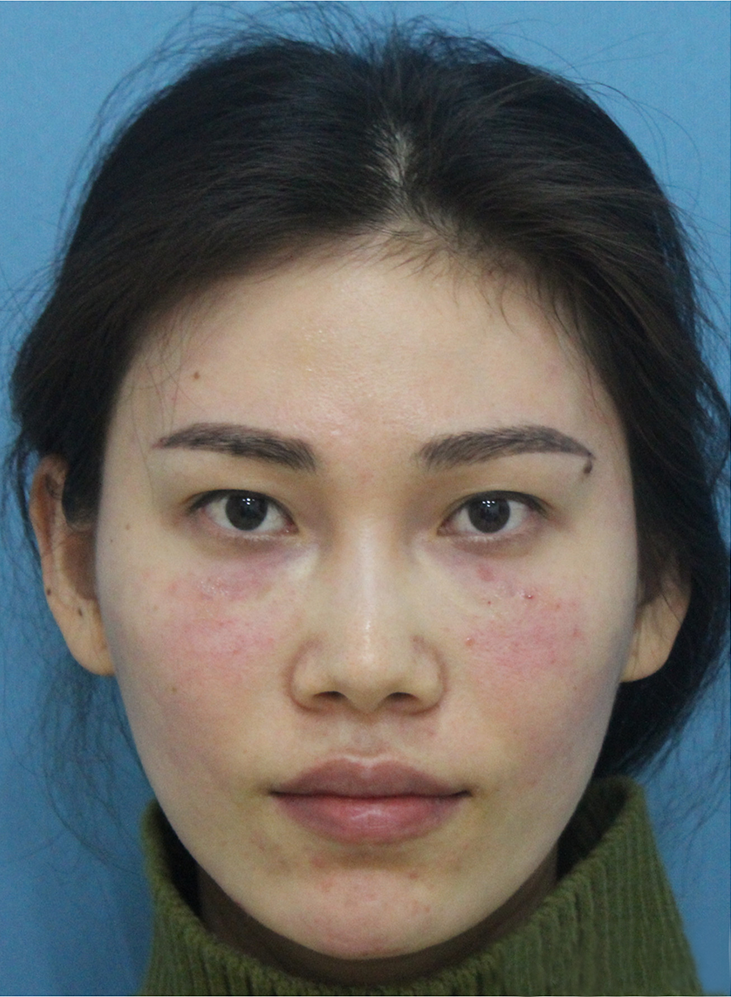

Supplement: ojaa053_suppl_Supplementary_Figures [file ojaa053_suppl_Supplementary_Figures.docx]
